# Vision Communication and Firm Quality Performance: The Mediating Role of Employee Involvement and the Moderating Effect of Leader Support

**DOI:** 10.3390/bs14100902

**Published:** 2024-10-07

**Authors:** Dan Ji, Jiankun Gong, Zheng Guo

**Affiliations:** 1 School of Humanity, Shanghai Jiao Tong University, Shanghai 200050, China; jidan@sjtu.edu.cn; 2 School of International and Public Affairs, China Institute for Urban Governance, Shanghai Jiao Tong University, Shanghai 200050, China; 3 Department of Media and Communication Studies, Faculty of Arts and Social Sciences, University of Malaya, Kuala Lumpur 50603, Malaysia; 4 International College, Krirk University, Bangkok 10900, Thailand; 5 Chinese Institute for Quality Research, Shanghai Jiao Tong University, Shanghai 200050, China; wshgz@163.com

**Keywords:** vision communication, quality performance, employee involvement, leader support, sensemaking

## Abstract

Vision communication (VC) is an important way for leaders to express their ideas about a blueprint of the future to convince employees that their work is valuable and meaningful in total quality management. This research studies the influence of vision communication (VC) on quality performance (QP) through the mediation of total employee involvement (EI) and the moderation of leader support (LS). With the use of Smartpls 3.0, the collected data based on 2996 samples in Shanghai were used to analyze the various constructs. The results show that both VC and EI affect QP and should be influenced by LS. EI was found to partially mediate the relationship between VC and QP. The results also demonstrate that EI can positively affect improvement in QP via stronger LS and that high-level employee involvement is positively associated with quality performance when leader support is high (vs. low). This research can be inferred as one of the very limited empirical analyses that explored the mediating impact of EI on VC and QP. In the quality management (QM) field, the exploration of the moderating effect of LS on the mediation of the EI between VC and QP can be viewed to be a significant theoretical finding. The findings will be instrumental in assisting managers and administrators in understanding the significance of vision communication and leader support in quality management practice.

## 1. Introduction

Organizations face a complex world and turbulent market environment [1]. In such a new economic environment, a firm’s flexibility and competitiveness are essential for the development of any organization [2]. Therefore, strengthening the organization’s competitive advantages has long been reckoned as one of the firm’s most important goals [3]. The extant literature recognizes total quality management (TQM) as an important approach to improving various aspects of performance and competitive advantages [4,5].

The past few years have shown the huge significance of TQM to firms’ economic and innovation outcomes. TQM affects the firm’s growth by the assumption that employees certainly pay attention to the quality of work if they are rendered with the ideas, tools, and training that are required for quality improvement [6]. However, only when the staff has strengthened their identification with the envisioned goal will they work harder to complete their regular tasks and try to achieve organizational goals.

We view the vision of quality management in organizations as an important factor that affects quality performance (QP) in TQM practice. The vision of quality management refers to the ultimate goal or collective future image of quality management [7]. Scholars believe that TQM breaks the conventional conventions and social relationships that employees rely on to complete their work and challenges the values that guide these practices. Vision communication (VC) is an important way for leaders to express their vision about TQM to convince employees that the vision is valuable. If leaders can clearly express unified images of the future, it will motivate followers to realize the vision and accomplish the TQM aim. However, there were limited findings in prior research on the relationship between vision communication and quality performance.

Many TQM scholars have also focused on the concept of employee involvement (EI); they stated that total employee involvement as an important part of TQM can have a positive effect on the financial performance of the organization [8]. Although there has been an increase in the TQM literature regarding various factors affecting EI, few studies have specifically examined the important role of EI as an independent factor affecting QP [9] and its role in the relationships between VC and QP. These research gaps limit the profundity of conceptual understanding and management experience of TQM.

Further, scholars have indicated possible research exploring the moderating impact of leader support (LS) as it is considered a significant precursor to QP in any quality improvement initiative [10,11]. The commitment of top management to disseminating vision can vary significantly between organizations, which may influence their QP in different ways. Therefore, further empirical investigation is required to establish conceptual generalization.

To address these research gaps, the current study examines the direct relationship between VC and QP and then explores the proposed VC-QP relationship through the possible mediation mechanisms of EI and the moderation of LS.

To test our hypotheses, we conducted research in Shanghai, obtaining responses from 2996 enterprises. Each of these size groupings implements TQM practice. This study uses partial least squares structural equation modeling (PLS-SEM) to analyze data and the hypotheses.

The results of this study provide valuable managerial insights, emphasizing the importance of vision communication in organizations, and also contribute to TQM research in several meaningful ways. First, it offers new insights into the TQM theory by empirically illustrating how VC affects QP from a sensemaking perspective. Second, it presents empirical support for the relationship between VC and QP from a moderated mediation perspective, which is a novel approach in OM research. Third, this study explores the mediating role of EI in the VC-QP relationship. Additionally, considering the discussion above, this study also investigates the extent to which LS moderates the effect of EI on QP.

The remainder of the paper is organized as follows: The next section provides an overview of the theoretical foundation and the literature on the relationships and interactions of vision communication, employees’ involvement, leader support, and quality performance, presenting the research hypotheses. Section 3 introduces the research methodology, followed by hypothesis testing and the reporting of results in Section 4. The final section discusses key findings, their implications, limitations, and opportunities for further research.

## 2. Literature Review and Hypotheses

### 2.1. Theoretical Foundation

Sensemaking refers to the process through which individuals assign meaning to issues or events that deviate from the expected state of the world developed by Weick et al. [12].

Sensemaking theory emphasizes the human-centered process of organizational behavior, which is an effective set of concepts and methods for studying how people construct their world and judge how people construct specific needs and behaviors. It is also one of the important theories of cognitive paradigms in the field of management. Sensemaking is an ongoing process that is observable in everyday organizational life [13], particularly during disruptive events or crises [14].

When TQM practice emerges as a significant event in organizational life, it can disrupt established meanings and prompt employees to question their understanding of organizational values [15]. This cognitive discomfort becomes a catalyst for the sensemaking process, leading employees to revise existing meanings and construct new ones related to TQM practices.

In TQM practice, sensemaking involves the construction and understanding of the value of quality management based on contextual signals or clues. It emphasizes the formation and reconstruction of individual meanings, which is a crucial micro-level behavior within organizations.

When an organization requires its members to form a unified understanding of TQM goals and participate in TQM practice, the process of quality management meaning construction becomes even more important. Maitlis divides stakeholders in the process of meaning construction into two entities: leaders and employees [16]. Leaders and managers in organizations have to interpret and communicate TQM practice standards and certification in ways that are meaningful to employees. The leader tends to use their power and channels for communication and interaction; their behavior in meaningful communication has a significant impact on the process. The main impact of employees in the process of meaning construction lies in the level of their involvement.

We view vision communication as a “meaning construction” used by leaders to make sense of TQM practice and to collectively construct meaning among employees to increase the effect of TQM. Therefore, we study how the leaders interpret and transfer their TQM vision to the employees to shape the TQM’s particular meaning, which leads to more employee involvement and then results in good quality performance.

### 2.2. Vision Communication and Quality Performance

Vision is deemed as abstract idealizations of organizational goals and strategies that focus on the future and are presented in an inspiring manner [17]. Extant research has proved that vision impacts organizational performance [18], effectiveness [19], growth [20], and leaders’ effectiveness [21].

For vision to have a significant impact on individuals and organizations, effective communication is essential [22]. The importance of vision communication lies in its ability to instill belief in a better future among employees, which is satisfying on its own [23].

Vision communication can be defined as the communication of a future state by leaders, often to mobilize followers toward collective action. Previous research in start-up businesses has shown that communicating a vision directly affects organizational performance and retail stores [24,25]. They also found a positive relationship between the extent to which a leader communicates the vision and levels of customer and follower satisfaction in the retail apparel industry [25]. Jing et al. demonstrated a positive relationship between vision communication and performance sharing in Australian retail pharmacies [18]. Gochmann et al. argued that vision communication can enhance the motivation and performance of followers, making it an essential tool for effective leadership [21]. However, there are very few studies have empirically investigated the possible association between vision communication and quality performance in organizations.

By vividly depicting and conveying a sense of what the future could look like through TQM, leaders can promote effective individual and collective action. An effective total quality management vision puts a strong emphasis on the shortcomings of the current situation and creates an ideal collective image for the future. The vision contains the collective identity and value evaluation of quality management, which facilitates the employees to understand the meaning of quality management practice. Vision communication will further affect the “choice” process of employee meaning construction, inducing employees’ involvement in quality management practice. Specifically, vision communication helps employees build a quality management narrative world. Vision communication conveys the essence, values, and objectives of the vision. It can help employees expand their cognition and provide them with a more comprehensive understanding of why the organization practices quality management, the direction of quality management, and their roles during the course. In the process of meaning building, employees tend to understand current quality management as part of the strategic development plan of the organization, rather than temporary actions taken by senior leaders, thereby enabling employees to recognize the significance of quality management. Vision communication also helps employees form positive psychological expectations in quality management practice. Vision provides a storyline from the current outlook to the future, triggering employees’ attention to the organization’s identity. When employees can connect their current change activities with the potential for future collective self, it generates hope and expectations for future work. In this way, the present and future are interconnected, and quality management will become a useful self-development process that can trigger a focused awareness of the meaning of work and a positive response to involvement.

Therefore, it is expected that communicating a vision will have positive effects on quality performance.

**H1.**  *Vision communication has a positive relation with quality performance*.

### 2.3. Vision Communication and Employee Involvement

Employee involvement has attracted academic attention since the 1990s. Employee involvement is generally defined as a process that allows employees to provide input into decisions that affect organizational performance and employee well-being [26].

Others have conceptualized involvement as a matter of degree or as a hierarchy of practices [27]. In some views of organizational researchers, employee involvement is composed of three essential variables, namely, involvement in decision-making, teamwork, and information communication [28].

Employee involvement, also known as employee participation, is a fundamental organizational communication practice that involves allowing, welcoming, and encouraging employees to contribute their voices and inputs in the management or structuring of the organizations [29].

Vision communication has been recognized as an important factor that can greatly influence employee involvement. A leader in an organization can create a vision and communicate it to employees so that they understand the meaning of their work and actively engage in it [30]. If employees’ personal goals align with the organization’s purpose, effective vision communication can enhance their intrinsic motivation [31]. Vision communication is an important tool that can activate employees’ self-awareness and understanding, thereby stimulating the necessary behaviors [32].

Qin argues that vision communication with employees in an organization is critical in increasing their willingness to engage with their work and achieve higher goals [33]. Effective vision communication motivates employees to support an organization’s long-term goals and empowers them to work toward achieving the ideal future state, which ultimately fosters employee engagement [34].

By communicating a long-term goal of TQM, leaders can interpret challenging tasks as stimulating, broaden perspectives on the tasks, construct the meaning of TQM, and provide the employees with a sense of identity and mission, which can encourage employees to be more enthusiastic in achieving the goal. When employees truly understand the meaning and value of the work, they are doing so through the communication of the vision with their leaders; they will believe that achieving the vision can realize their value and meet their needs, and will then take proactive actions beyond the prescribed job content [35]. Based on the above, the following hypothesis can be proposed:

**H2.**  *Vision communication has a positive relation with employee involvement*.

### 2.4. Mediation of Employee Involvement

Human resources are considered to be a pivotal factor in the competitive advantage of every organization. Empowering human resources by promoting their involvement in organizational decision-making and job affairs can increase organizational effectiveness in practice [36].

Employee involvement includes empowering lower-level employees to engage and take responsibility for making organizational decisions. It enables employees to align their interests with those of the organization, which is the primary goal of employee involvement.

In recent decades, employee involvement has gained significant attention in management, and various participative practices/approaches have been implemented in multiple workplaces with varying results [9]. Armstrong has demonstrated that involvement ensures that employees can influence management decisions and improve organizational performance [37]. Naqshbandi et al. demonstrated the mediating role of employee involvement climate in the empowering leadership-inbound open innovation link [38]. The extant literature also indicates that employee involvement is positively associated with higher organizational commitment and satisfaction levels in European [39] and Asian contexts [40,41].

Transforming the vision of a total quality organization to reality requires a complete change in prevailing attitudes and culture within the company. This change must cascade from top management to the shop floor and must be permanent, consistent, and visible [42]. Effective implementation of TQM practices requires the commitment of all levels of staff, making employee involvement and participation essential for the success of quality initiatives [43].

Vision communication in quality management is the mechanism of dialogue among workers to exchange information and ideas, which satisfies employees’ higher needs and encourages them to improve their job performance and benefit the whole organization. In TQM implementation vision communication will reduce employee misunderstanding about the goal of TQM and encourage the employee to participate in the course. Once the organization makes employees understand the meaning of the task, the employees will become involved, and they will better understand the rationale behind the TQM and devote more energy to their work to generate positive outcomes.

Based on the above arguments, the following hypothesis can be postulated:

**H3.**  *Employee involvement has a positive relationship with quality performance*.

**H4.**  *Employee involvement mediates the relationship between vision communication and quality performance*.

### 2.5. Moderation of Leader Support

Leader support is defined as the perceived level of support from supervisors by employees. The degree to which employees receive support is vital to their performance and satisfaction [44]. In this study, leader support is considered a necessary relational condition in the workplace, representing the leader’s series of actions to help members, such as by providing job resources like information.

Several studies have theorized and examined the role of leader support in prompting proactive behavior. Amabile et al. established that leader support is proposed to be a key feature of the work environment for creativity [45]. Cheung and Wong demonstrated that leader support may provide different work environments in which transformational leaders can influence the creativity of their followers [46]. A central argument is that having support from leaders fosters a higher sense of self-determination [47] and enhances employees’ sense of competence and willingness to initiate future-focused change [48].

Amabile et al. indicated that employees who perceived the adequacy of useful job feedback from the leader might have their beliefs about the intrinsic value of the work that they have undertaken affected [49]. When the level of support from team leaders is high, the employees will be encouraged to participate in quality management work and improve quality performance. Therefore, effective support from team leaders will make team members more willing to engage in the work and enhance their self-efficacy to promote quality performance. This not only enables team members to immerse themselves in their work but also fosters a trusting and supportive work environment, effectively motivating their enthusiasm and enhancing their focus on the enterprise’s quality development [50].

In departments with a supportive leadership atmosphere, employees can receive more help and guidance from department managers. Therefore, their role clarity is higher, and they successfully establish the meaning of their work and can better implement quality management practices. However, in departments with poor supportive leadership atmospheres, the majority of employees lack the support, guidance, and assistance of management personnel and do not know how to play their job roles well. Therefore, their perceived role ambiguity is more likely to lower the quality of their performance.

Based on these arguments, the following hypothesis is derived:

**H5.**  *LS positively and significantly moderates the relationship between EI and QP*.

**Figure 1:**
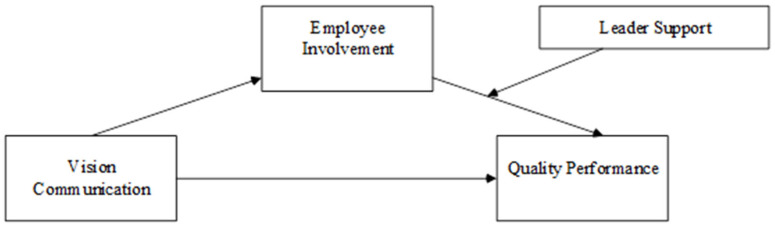
Theoretical framework.

Based on the arguments above, we propose the research model below (see Figure 1).

## 3. Method

### 3.1. Sample and Procedure

In order to ensure the confidentiality of the responses, a letter of permission for conducting research was obtained from the Shanghai Market Bureau. A cross-sectional quantitative research method was employed to collect data for this study. The approach is commonly used in empirical investigations, due to the ease of data collection at a single point in time, as opposed to a longitudinal design. This study was conducted in Shanghai between June and August 2022 involving 10,000 local small and medium enterprises. Out of the 10,000 questionnaires administered, a total of 2996 enterprises were evaluated, resulting in a response rate of 29.96%. Sample characteristics are described in Table 1. The questionnaires were delivered to the staff who were in the quality management department.

### 3.2. Measures

To test the proposed hypotheses, a survey questionnaire was developed. The items for all constructs were adopted from the previous literature and then tailored to the specific research context. Instrument items for constructing vision communication were adapted from Kantabutra and Avery [25]. Items of employee involvement were adapted from Locke et al. [51]. Leader support items were adapted from Yukl [52]. Quality performance items were adapted from Patyal and Koilakuntla [53]. Survey items are listed in Table 2. All construct items were anchored on a 5-point Likert scale ranging from 1 for strongly disagree to 5 for strongly agree. The reliability and validity of the items were assessed using structural equation modeling.

Prior to the survey administration, a pilot test was conducted to ensure the relevance and appropriateness of the questionnaire for the target respondents. A total of 50 questionnaires were distributed face-to-face to the owners/managers of the enterprises, and 39 responses were received without any feedback on the questionnaire, indicating its clarity and ease of understanding, thus confirming its content validity. Additionally, Cronbach’s alpha was calculated to assess the internal reliability of items, and all the scales demonstrated an acceptable alpha value of >0.7 [54].

### 3.3. Common Method Variance Bias

The current study adopts a quantitative research approach, collecting data from a single source. Previous studies have highlighted the potential issue of common method variance when data are collected with a single source. Therefore, it is important to test for common method variance issues before conducting inferential analysis. To address this concern, Harman’s single-factor test is employed [55]. The criterion for this test is that the maximum covariance explained by the first factor should not exceed 50% [55]. The results of Harman’s single factor test revealed that the maximum covariance explained by a single factor was only 18% which is below the threshold value of 50%, confirming that this study is free from common method variance bias and is suitable for structural equation modeling.

## 4. Data Analysis

The present study proposes an amalgamated model to investigate the relationship between vision communication and quality management performance. To analyze the data, this study employs the structural equation modeling technique (SEM), which is a statistical approach for testing and estimating causal relations using a combination of statistical data and qualitative causal assumptions [56]. The analysis follows a two-stage approach, consisting of a measurement model and a structural model in line with Rahi [57]. The measurement model assesses construct reliability, validity, indicator reliability, convergent validity, and discriminant validity. The structural model estimates the path and their significance levels. Smart PLS 3.27 software is utilized for structural equation modeling [58].

### 4.1. Measurement Model

In the measurement model analysis, this study employs structural equation modeling using the partial least squares (PLS) methodology with Smart PLS 3.0 to evaluate the quality of the measurement tool and test the hypotheses. PLS is deemed appropriate considering the sample size (n = 2996), the focus on each path coefficient, and the emphasis on variance explained rather than overall model fit.

To ensure construct reliability and validity, this study tests reliability, convergent validity, and discriminant validity. Reliability is assessed through Cronbach’s alpha, with values exceeding 0.70 indicating statistical reliability. One item (LS2) with a low item-total correlation is excluded. Table 3 displays Cronbach’s alphas for all eight constructs which all surpass the recommended level of reliability.

Convergent validity, which measures the extent to which multiple items effectively measure the same construct, is evaluated using factor loadings, composite reliability (CR), and average variance extracted (AVE). Table 3 demonstrates that factor loadings exceed the minimum requirement of 0.70, indicating satisfactory convergent validity. Composite reliability exceeds the minimum value of 0.70, and the AVE for each construct surpasses the minimum value of 0.50, further confirming convergent validity.

Discriminant validity, which assesses whether two constructs are statistically different, is evaluated using the Fornell–Larcker criterion and HTMT. Table 4 represents the diagonal line of elements reflecting AVE square roots with constructs below. By comparing the diagonal and off-diagonal lines, distinguishable validity can be determined, as the diagonal line values in the line and column are high.

The values of the HTMT criterion are lower than the required threshold value of 0.9 [59]; thus, in referring to the HTMT criteria, there were no discriminant validity issues for inter-construct correlations of 0.898 and lower. To further validate this finding, we also examined the HTMT inference and found that it was statistically different from 1 [60].

### 4.2. Structural Model

To assess collinearity in the structural model, as recommended by Hair et al. [61], the presence of multicollinearity was examined using the variance inflation factor (VIF). A VIF value of five or above indicates the presence of multicollinearity. In this study, the VIF values were around two or below, indicating no issue with multicollinearity.

**Figure 2:**
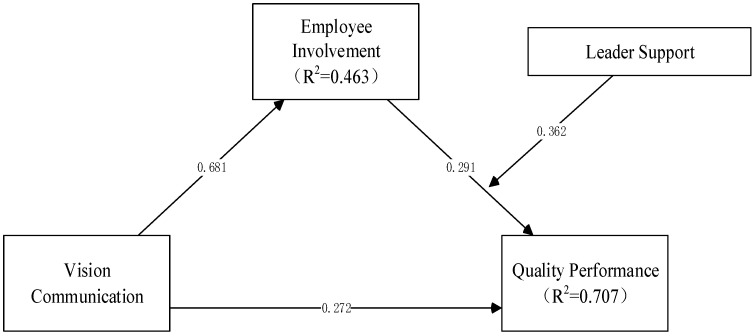
Structural model results (SEM).

The structural models for this research are presented in Figure 2, where R^2^ represents the value for each endogenous and predicted latent variable. Previous studies have established threshold values of R^2^, with 0.75, 0.50, and 0.25 representing substantial, moderate, and weak relationships, respectively [60,62] For the dependent variable, quality performance (QP), the R^2^ value is 0.707, indicating that it explains 70.7% of the variance in quality performance. The present study found that 46.3% and 70.7% of the variations in QP can be, respectively, attributed to the independent variables VC and EI.

According to Cohen’s criterion [63], the *f*^2^ values of 0.02, 0.15, and 0.35, respectively, represent small, medium, and large effects of the exogenous latent variable. The *f*^2^ values for VC → QP, EI → QP, and VC → EI were 0.107, 0.109, and 0.864, indicating small-to-large effect sizes.

Additionally, the standardized root mean squared residual (SRMR), which indicates the approximate fit of the model, was assessed. The SRMR measures the difference between the observed correlation and the model-implied correlation matrix [64]. A model is viewed as a good fit if it has an SRMR value less than or equal to 0.08 [65]. The SRMR value of this study was 0.075, confirming that our model meets the criteria for fit based on its SRMR value.

Path analysis was conducted using Smart-PLS 3 software with bootstrap sampling to obtain the *β* and *p*-values. The results provide support for the model’s direct, indirect, mediation, and moderation effects.

#### 4.2.1. Direct Effect

The results presented in Table 5 and Figure 2 show that VC has a positive effect on QP (*β* = 0.273 at *p*-value < 0.01) and EI (*β* = 0.681 at *p*-value < 0.01). It also indicated that the variable EI has a positive effect on QP (*β* = 0.296 at *p*-value < 0.01). H1, H2, and H3 were supported.

#### 4.2.2. Mediation Effect

To examine the mediation effect, this study assessed the role of EI in the VC-QP relationship. The result indicated that EI (*β* = 0.198 at 95% confidence interval = (0.231–0.275)) mediates the nexus between VC and QP, confirming the indirect hypothesis H4. The strength of mediation was evaluated using the variance accounted for (VAF) method. Since the indirect effect is significant in this study, VAF is calculated as mediating effect/total effect (mediating effect + direct effect), which implies that VAF = 0.202/0.202 + 0.273; thus, VAF = 0.4252. According to Hair et al. [54], a VAF value between 0.20 and 0.80 indicates partial mediation. Therefore, it can be concluded that EI partially mediates the relationship between VC and QP.

#### 4.2.3. Moderation Effect

**Figure 3:**
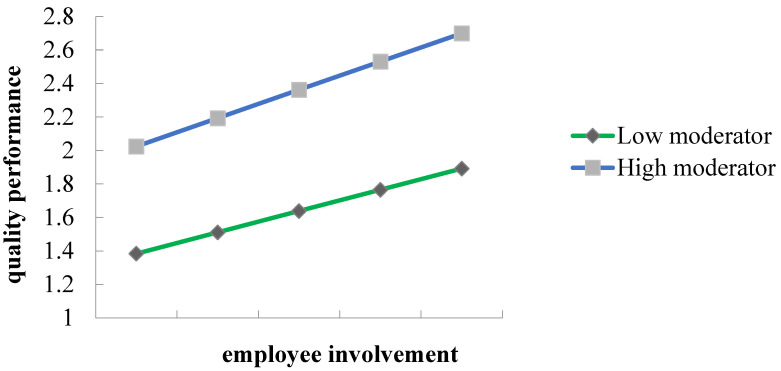
Moderation (EI * LS → QP).

Furthermore, this study investigated the moderation effect of leader support (LS) on the relationship between employee involvement and quality performance. The results in Table 5 indicate that leader support positively moderates this relationship (*β* = 0.042 at *p*-value = 0.015). Thus, hypothesis H5 is accepted. The summary of the hypothesis testing in listed in Table 6. The interaction graph in Figure 3 illustrates that leader support strengthens the relationship between employee involvement and quality performance. The graph shows that if enterprises’ quality management practices more leader support, the moderation effects will be higher, and QP will be enhanced. Lastly, a PLSpredict technique was performed to determine the predictive relevance (Q^2^ predict) of the model. The Q^2^ predict values for all endogenous constructs in Table 6. exceeded zero (0.393–0.429), demonstrating the model’s predictive relevance, and supporting the model’s predictive capacity on the endogenous variables [66]. Therefore, it is surmised that the current model involving visual communication and employment involvement as the predictors possesses the capacity to predict quality performance in the given context.

The acceptable range for the SRMR index is between 0 and 0.08. As seen in Table 7, the SRMR values are within the threshold for the saturated model and slightly over it for the estimated model. A value greater than 0.9 for NFI denotes a good fit; hence, the above values indicate a strong model. Thus, they are believed to be substantively significant for the study’s implications.

## 5. Discussion

This study attempted to explore the relationship between vision communication and enterprise quality performance. While previous research has explored factors affecting total quality management performance, the interaction between VC, EI, LS, and QP remains understudied. This study contributes to the existing knowledge by examining the mediating role of EI in the VC and QP nexus and the moderating effect of LS in the relationship between EI and QP.

All five hypotheses proposed were found to be statistically significant.

First, the findings support H1, indicating a positive and significant relationship between vision communication and quality performance.

A clear vision statement is a key factor for enterprises to achieve sustainable development [67]. However, the extant literature has not extensively discussed the mechanisms of the organization’s vision communication that promote the enterprise’s quality performance. This study demonstrates that VC positively affects QP in Chinese enterprises, serving as a motivational factor for employees and contributing to organizational performance. These results align with previous studies conducted in emerging markets [18] and support the applicability of sensemaking theory in explaining the relationship between VC and QP.

Second, we demonstrated that VC has a direct relationship with EI. Leaders rely on their visions to communicate strategic goals, beliefs, values, and statements of purpose to effectively motivate their employees [68]. Drawing upon sensemaking theory, this study confirms that VC has a positive influence on employees’ proactive performance. These results are consistent with previous research that highlights the positive effect of leaders’ vision communication on employees’ performance.

Furthermore, this study examined the direct link between EI and QP and found a positive influence of EI on QP. Prior studies indicate that EI positively influences the contribution of TQM to the improvement of business performance [69,70]. This finding provided empirical support for the managers to focus on the relationship between employee involvement and quality performance from sensemaking theory. Sensemaking theory is particularly relevant in understanding the implementation of total quality management [71], as employees engage in cognitive processes to comprehend the meaning and impact of TQM on them and the organization. Based on sensemaking theory, this study supports the notion that employee involvement is a valuable resource for organizational success, as employees collectively make sense of the event.

Our research design is similar to that of previous studies that examined the contribution of the mediating role of EI. The findings confirmed that EI partially mediates the relationship between VC and QP. The results are consistent with a previous study conducted by Tortorella et al. [72], which reported a positive mediating effect of employee involvement on the relationship between Industry 4.0 adoption and operational performance improvement.

Lastly, the moderating effect of leader support on the relationship between EI and QP is examined. We found that even though the relationship between employee involvement and quality performance was positive, the strength of the relationship varied depending on leader support. Leader support plays a crucial role in this relationship as it positively moderates it. EI had a moderate positive relationship with QP when employees had high leader support. This finding is consistent with other studies that found that moderators such as high member turnover [73] and low-quality employee relations [74] neutralized EI and performance relationships.

## 6. Conclusions and Implications

### 6.1. Conclusions

This study adopts a cross-sectional perspective to examine the direct and indirect effects of vision communication and employee involvement on total management quality performance in China. By considering the mediating role of employee involvement, this study incorporates employee involvement in total management practice and highlights the significance of LS in the relationship between employee involvement and quality performance. The model was empirically tested using SEM analysis on a sample of 2996 Chinese enterprises. The findings demonstrated the positive impact of VC on EI and QP, with EI partially mediating the relation between VC and QP. Furthermore, leader support positively moderates the relationship between employee involvement and quality performance in China.

### 6.2. Theoretical Implication

This study has important theoretical implications. First, our study extends the sensemaking theory. We illustrate the important role of vision communication in sensemaking during TQM. Sensemaking is a process through which organizational actors construct meanings by extracting, interpreting, and acting upon cues from their environment [75]. According to Weick [76], sensemaking is, importantly, an issue of language, talk, and communication. Communication plays a crucial role in sensemaking, and the literature on it is diverse and fragmented. This study contributes to the literature by examining the impact of VC on QP in China, demonstrating its influence on the processes and outcomes of sensemaking.

Second, this study reveals the importance of employee involvement as an important link between VC and QP. Employee involvement is a vital element in TQM and is strongly related to the success of continuous improvement [77]. While previous studies have examined various antecedents of employee involvement such as empowerment, financial and non-financial rewards, and training that promotes improvements [78], few have explored it as a mediator between VC and QP. This study fills this gap and provides empirical evidence of the mediating role of employee involvement in the VC-QP nexus.

Lastly, this study sheds light on the moderating impact of leader support on the relationship between employee involvement and quality performance. It highlights that leader support enhances the impact of employee involvement on quality performance, emphasizing the role of leaders as enablers or relationship moderators [79].

### 6.3. Managerial Implications

At the managerial level, the findings have practical implications. First, managers should recognize the significant effect of VC on quality performance and ensure that the vision and accompanying values of TQM are effectively communicated to employees during implementation. Managers can unite management and employees by vision communication during the implementation of TQM.

Second, the results prove the positive relationship between employee involvement and quality performance. Organizations are encouraged to adopt and develop reliable employee involvement programs in the TQM practice to enhance performance.

Furthermore, our study also suggests that employee involvement is so important that leaders can play a crucial role in encouraging and motivating employee participation in TQM practice for better outcomes.

## 7. Limitations and Future Research

This research still has several limitations that should be addressed in future studies. First, the cross-sectional design limits the understanding of the factors affecting quality performance over time. Future studies could employ longitudinal designs to understand the factors affecting quality performance in TQM practice including VC, EI, LS, and QP.

Second, the model does not account for potential external or internal factors that may influence these relationships. Factors such as organizational culture, industry type, economic conditions, and company size could moderate the effects of VC, EI, and LS on QP. Additionally, individual-level factors such as employee demographics, job roles, and tenure may also play a role in shaping these relationships. Including such factors in future research would offer a more comprehensive understanding of the dynamics at play and improve the generalizability of the findings across different contexts.

Third, this study is based on the context of one region. Future studies could examine the implementation of quality management in other cultural contexts.

## Figures and Tables

**Table 1:** Sample characteristics.

Demographic Variables	Item	Frequency	Percentage
Type of business	State-owned business	328	10.95%
	Private-owned business	2120	70.76%
	Others	548	18.29%
Number of employees	Less than 300 employees	2535	84.6%
	300–1000 employees	294	9.8%
	More than 1000 employees	167	5.6%
Annual revenue	Less than 3 million	457	15.25%
	3 million to 20 million	886	29.57%
	20–400 million	1249	41.7%
	More than 400 million	404	13.48%

**Table 2:** Survey items.

Items		Source
**Vision Communication**		
VC1	My manager talks with me about the vision of quality management.	Kantabutra and Avery [25]
VC2	My manager mentions the vision of quality management in staff meetings regularly.	
VC3	My manager talks with me about the vision of quality management using technology-mediated channels.	
**Employee Involvement**		
EI1	The company’s decisions are influenced by my views.	Locke, Schweiger, and Latham [51]
EI2	I have a word in selection and training decisions.	
**Leader Support**		
LS1	My leader demonstrates sympathy and support when I am feeling worried or upset.	Yukl [52]
LS2	My leader provides encouragement and support when I am faced with difficult and stressful tasks or responsibilities.	
LS3	My leader offers advice and assistance whenever I encounter challenging tasks or problems.	
**Quality Performance**		
QP1	There have been notable improvements in the delivery of products and services within our organization over the past three years.	Patyal and Koilakuntla [53]
QP2	Over the past 3 years, the cost of scrap and rework as a percentage (%) of sales has decreased in your organization.	
QP3	Over the past 3 years, a reduction in cycle time, from receipt of raw materials to shipment of finished products in your organization.	
QP4	Over the past 3 years, customer satisfaction with the quality of products and services has shown an increase in your organization.	

**Table 3:** Assessment of construct reliability and convergent validity.

	Items	Factor Loading	AVE (>0.5)	Composite Reliability (>0.6)	Cronbach’s Alpha (>0.6)
Value Communication (VC)	VC1	0.781	0.626	0.834	0.703
	VC2	0.782			
	VC3	0.811			
Quality Performance (QP)	QP1	0.822	0.506	0.801	0.754
	QP2	0.857			
	QP3	0.475			
	QP4	0.808			
Employee Involvement (EI)	EI1	0.909	0.837	0.911	0.805
	EI2	0.920			
Leadership Support (LS)	LS1	0.873	0.768	0.868	0.702
	LS3	0.882			

**Table 4:** Analysis of Fornell–Larcker discriminant validity.

	EI	LS	QP	VC
EI	0.915			
LS	0.777	0.876		
QP	0.344	0.362	0.712	
VC	0.681	0.736	0.344	0.792

**Table 5:** Analysis of HTMT discriminant validity.

	EI	LS	QP	VC
EI				
LS	0.820			
QP	0.844	0.738		
VC	0.898	0.874	0.891	

**Table 6:** Summary of the hypothesis testing.

Hypothesis	Paths	*β*	(STDEV)	*t*-Value	*p*-Value	Confidence Interval	Decision	Q^2^
H1	VC -> QP	0.273	0.017	15.914	0.000	(0.231 0.275)	Supported	0.429
H2	VC -> EI	0.681	0.011	62.416	0.000	(0.663 0.699)	Supported	0.393
H3	EI -> QP	0.296	0.020	16.112	0.000	(0.343 0.400)	Supported	
H4	VC -> EI- > QP	0.202	0.013	15.471	0.000	(0.231 0.275)	Supported	
H5	EI * LS -> QP	0.042	0.009	4.940	0.000	(0.019 0.047)	Supported	

**Table 7:** Model fit summary.

	Saturated Model	Estimated Model
SRMR	0.078	0.103
d_ULS	0.274	0.481
d_G	0.153	0.202
Chi-Square	2726.075	3231.947
NFI	0.979	0.938

## Data Availability

Data can be provided upon reasonable request for academic purposes only from the corresponding author.
